# Danger-Associated Peptide Regulates Root Immune Responses and Root Growth by Affecting ROS Formation in *Arabidopsis*

**DOI:** 10.3390/ijms21134590

**Published:** 2020-06-28

**Authors:** Yanping Jing, Nuo Shen, Xiaojiang Zheng, Aigen Fu, Fugeng Zhao, Wenzhi Lan, Sheng Luan

**Affiliations:** 1 College of Life Sciences, Northwest University, Xi’an 710069, China; jingyanping1989@163.com (Y.J.); xjzheng1985@berkeley.edu (X.Z.); aigenfu@nwu.edu.cn (A.F.); 2 Nanjing University-Nanjing Forestry University Joint Institute for Plant Molecular Biology, College of Life Sciences, Nanjing University, Nanjing 210093, China; SN19921112520@163.com (N.S.); fgzhao@nju.edu.cn (F.Z.); 3 Department of Plant and Microbial Biology, University of California, Berkeley, CA 94720, USA

**Keywords:** *Arabidopsis*, DAMPs, ROS, root immune responses, root growth

## Abstract

Plant elicitor peptides (Peps) are damage/danger-associated molecular patterns (DAMPs) that are perceived by a pair of receptor-like kinases, PEPR1 and PEPR2, to enhance innate immunity and induce the growth inhibition of root in *Arabidopsis thaliana*. In this study, we show that PEPR1 and PEPR2 function vitally in roots to regulate the root immune responses when treating the roots with bacterial pathogen *Pst DC3000*. PEPR2, rather than PEPR1, played a predominant role in the perception of Pep1 in the roots and further triggered a strong ROS accumulation—the substance acts as an antimicrobial agent or as a secondary messenger in plant cells. Consistently, seedlings mutating two major ROS-generating enzyme genes, *respiratory burst oxidase homologs D* and *F* (*RBOHD* and *RBOHF*), abolished the root ROS accumulation and impaired the growth inhibition of the roots induced by Pep1. Furthermore, we revealed that botrytis-induced kinase 1 (BIK1) physically interacted with PEPRs and RBOHD/F, respectively, and served downstream of the Pep1-PEPRs signaling pathway to regulate Pep1-induced ROS production and root growth inhibition. In conclusion, this study demonstrates a previously unrecognized signaling crosstalk between Pep1 and ROS signaling to regulate root immune response and root growth.

## 1. Introduction

Plants, being sessile, have evolved a sophisticated innate immune system based on the perception of pathogen-associated molecular patterns (PAMPs) by the plasma membrane-localized pattern recognition receptors (PRRs) to trigger the defense response, which is also called pattern-triggered immunity (PTI) [1,2,3,4]. In *Arabidopsis thaliana*, the leucine rich repeat receptor-like kinase (LRR-RLK), FLS2 (flagellin-sensitive 2), is one of the best studied PRRs that recognizes the conserved 22-amino-acid epitope of bacterial flagellin (flg22) [5,6]. In addition to PAMPs, plants also release specialized molecules, known as damage- or danger-associated molecular patterns (DAMPs) to trigger the defense response [3,7]. In *Arabidopsis*, a well-recognized DAMP is a family of plant elicitor peptides (Peps), which are derived from the C-terminal regions of their precursor proteins, PROPEPs, and are perceived by the receptor-like kinases PEPR1 and PEPR2 [8,9]. Among the two receptors, PEPR1 and PEPR2 express at similar levels in plant leaf and root tissues, but the functions between two receptors are different [10]. PEPR1 mainly functions in leaves to regulate stomatal immunity, while the binding of Peps to PEPR1 elicits strong immune responses in a similar way to flg22-FLS2 signaling in plant leaves. As a result, the Peps-PEPRs system is termed as an amplifier of flg22-FLS2 signaling [11,12]. However, in the root tissues, PEPR2 is the dominant receptor for Peps and the binging of Peps to PEPR2 elicits a strong root growth inhibition [13].

Most studies on plant immunity have focused on microbial responses in leaf tissues. The root tissues also maintain close ties to their biotic environment. The rhizosphere pathogenic communities are larger than those surrounding the leaves and constantly threaten plant health [14,15]. To respond to the pathogen attacks, the plant roots may develop sophisticated mechanisms to perceive these pathogens carrying potential PAMPs and trigger the immune response to improve plant fitness. However, root immunity is poorly characterized as compared to studies on leaf immunity, due to the low accessibility of both the pathogens and the host organs underground [14,16]. Several studies have indicated that the application of flg22 or Peps triggered a series of responses in roots, including elevated cytosolic Ca^2+^ levels, reactive oxygen species bursts, callose deposition, and defense-related gene expression, implying that roots may share similar immune responses with those in leaves [10,16,17,18,19].

The reactive oxygen species (ROS) acts as an antimicrobial agent or as a secondary messenger in plant cells to trigger additional immune responses to improve plant resistance [20,21,22]. There are mainly two types of ROS, including superoxide anion (O_2_^−^) and hydrogen peroxide (H_2_O_2_), which accumulate in plant cells [22]. The plasma membrane localized nicotinamide adenine dinucleotide phosphate (NADPH) oxidases, also known as respiratory burst oxidase homologs (RBOHs), are major ROS-generating enzymes responsible for ROS production in plant cells [23,24]. In *Arabidopsis*, there are ten members in the RBOH family, termed as RBOHA to RBOHJ [25]. RBOHD and RBOHF function more importantly than other members in apoplastic ROS generation during abiotic and biotic stimuli [24,26,27]. In the previous results, the flg22-FLS2 and Peps-PEPRs signaling were shown to trigger ROS production by activating the RBOHD in leaves. The botrytis-induced kinase 1 (BIK1) acted downstream of flg22-FLS2 and Peps-PEPRs signaling to interact and phosphorylate the RBOHD [21,24,28].

In our efforts to dissect the immune response in plants, we previously found that Peps-PEPRs signaling played a role in regulating stomatal immunity and triggering root growth inhibition [12,13]. However, in the current study, we show that PEPR1 and PEPR2 are required to trigger ROS accumulation when the roots are threatened by pathogens. Pep1-PEPR2, rather than Pep1-PEPR1 signaling, triggers a strong ROS accumulation, which implies that PEPR2 function more importantly than PEPR1 in roots to regulate the immune responses. Furthermore, we reveal that Pep1 triggers strong ROS production, which requires RBOHD and RBOHF. The two proteins also function vitally to mediate Pep1-induced root growth inhibition. Finally, we found that BIK1 functions downstream of Pep1-PEPRs signaling and physically interacts with PEPRs and RBOHD/F to regulate ROS production and root growth inhibition. This study thus demonstrates a previously uncovered signaling pathway between Pep1 and ROS signaling in plant roots.

## 2. Results

### 2.1. PEPRs Is Required for Activating the Roots’ Innate Immune Responses

**Figure 1:**
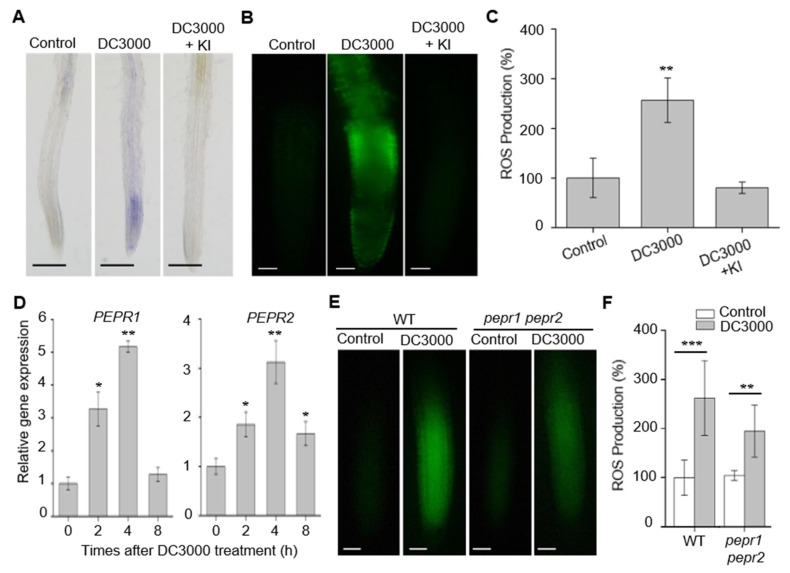
PEPRs are required to regulate the root immunity. (**A**) NBT staining of superoxide (O_2_^−^) anion in wild type (WT) roots. Six-day-old seedlings were incubated in half-strength Murashige and Skoog (MS) liquid medium (Control), *Pst* DC3000 (DC3000) solution (initial OD_600_ of 0.02), or DC3000 solution (initial OD_600_ of 0.02) and added to 100 μM KI for 18 h. The roots were stained with 1 mM NBT solution for 60 min and photographed under microscopy. Bars = 200 μm. (**B**) H_2_DCF-DA staining of H_2_O_2_ in WT roots. Six-day-old seedlings were incubated in half-strength MS liquid medium (Control), DC3000 solution (initial OD_600_ of 0.02), or DC3000 solution (initial OD_600_ of 0.02) and added to 100 μM KI for 18 h. The roots were stained with 30 μM H_2_DCF-DA solution for 10 min and photographed under fluorescence microscopy (Olympus, BX53). Bars = 100 μm. (**C**) The statistical analysis of ROS production in (**B**). The relative ROS production of each treatment was normalized to control the wild type (100%). Data are means ± SD from three independent experiments (*n* = 20). (**D**) Quantitative RT-PCR analysis of mRNA levels of *PEPR1* and *PEPR2* in the wild type roots, treated with DC3000 solution (initial OD_600_ of 0.02) for 0, 2, 4, and 8 h, respectively. The expression level of control (0 h) was set as 1.0 and the DC3000 treatment levels were normalized to the control level. Data are means ± SD (*n* = 3). (**E**) H_2_DCF-DA staining of H_2_O_2_ in WT and *pepr1 pepr2* roots. Six-day-old seedlings were incubated in half-strength MS liquid medium (control) and DC3000 solution (initial OD_600_ of 0.02) for 18 h. The roots were stained with 30 μM H_2_DCF-DA solution for 10 min and photographed under fluorescence microscopy (Olympus, BX53). Bars = 100 μm. (**F**) The statistical analysis of ROS production in (**E**). The relative ROS production of each treatment was normalized to control the wild type (100%). Data are means ± SD from three independent experiments (*n* = 24). Asterisks in (**C**), (**D**), and (**F**) indicate statistically significant differences compared with the controls in each genotype (Tukey’s test, * *p* < 0.05, ** *p* < 0.01, *** *p* < 0.001).

Plant roots are surrounded by a large community of pathogenic bacteria; however, root immunity is poorly characterized. To evaluate whether roots share the similar immune responses as leaves, we firstly soak-incubated the wild type roots with pathogen *Pseudomonas syringae* p.v. tomato DC3000 (*Pst* DC3000) suspension, followed by examining O_2_^−^ and H_2_O_2_, the two types of ROS as early defense markers [23]. By using the nitroblue tetrazolium (NBT) staining of O_2_^−^, we found that *Pst* DC3000 treatment strongly induced the accumulation of O_2_^−^ in the wild type roots, as shown in Figure 1A. We further stained the roots by using 2′, 7′-dichlorodihydrofluorescein diacetate (H_2_DCF-DA) to detect H_2_O_2_. Similar to O_2_^−^, the *Pst* DC3000 treatment increased the H_2_O_2_ content by 2.6-fold in the wild type plants, as shown in Figure 1B–C. However, such an increase was inhibited by 100 μM potassium iodide (KI), as shown in Figure 1A–C, a scavenger of ROS [29]. These results indicate that roots activate ROS signaling during pathogen infection, which is consistent with the previous studies [19].

The *Arabidopsis* PEPR1 and PEPR2 are stereotypical LRR-RLKs reported to regulate plant immunity, as the disruption of the genes encoding these receptors compromise plant immunity and enhance pathogen proliferation in plant leaves [8,9,30]. Both of the genes express in leaf and root tissues [10]. To evaluate whether PEPR1 and PEPR2 take part in root immunity, we first resorted to the real-time quantitative reverse transcription PCR (qRT-PCR) assay to measure the mRNA levels of the *PEPR1* and *PEPR2* over a time course upon *Pst* DC3000 treatment. We found that the expression of *PEPR1* and *PEPR2* were induced in roots after pathogen treatment, as shown in Figure 1D, consistently with the leaf responses, as shown in Figure S1, implying that *PEPR*s may be involved in plant immune responses in both root and leave tissues. Furthermore, we inoculated the seedlings with a *Pst* DC3000 bacterial suspension, followed by an examination of ROS (H_2_O_2_) production. We found that the root structure in the WT and *pepr1 pepr2* seedlings did not show significant difference upon *Pst* DC3000 treatment, as shown in Figure S2. The roots from the mutant plants *pepr1 pepr2* showed increased ROS under *Pst* DC3000 treatment, but the ROS levels were much lower than those in wild type roots, as shown in Figure 1E–F. Taken together, these results suggest that PEPRs are required to regulate root immunity.

### 2.2. Pep1–PEPR2 System Strongly Elicits the ROS Production in Root

**Figure 2:**
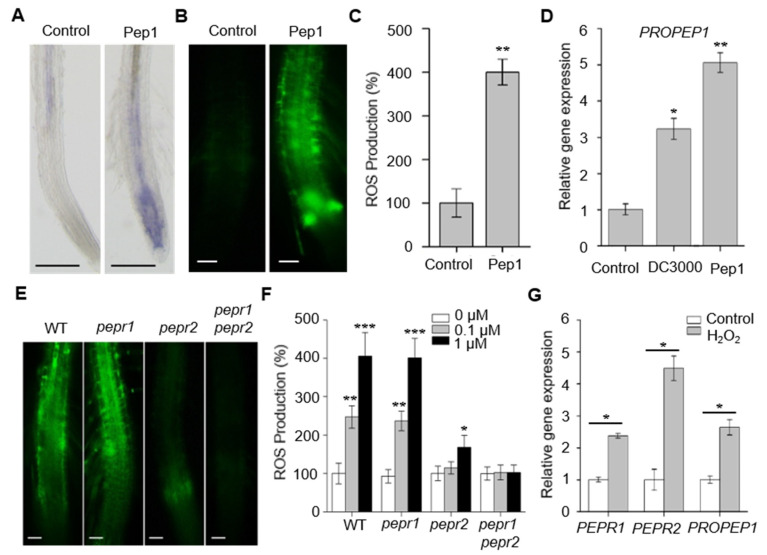
Pep1-PEPRs system activates the ROS signaling in root. (**A**) NBT staining of superoxide (O_2_^−^) anion in wild type (WT) roots. Six-day-old seedlings were incubated in half-strength MS liquid medium with or without (Control) 1 μM Pep1 for 18 h, the roots were stained with 1 mM NBT solution for 60 min and photographed under microscopy. Bars = 200 μm. (**B**) H_2_DCF-DA staining of H_2_O_2_ in WT roots. Six-day-old seedlings were incubated in half-strength MS liquid medium with or without (Control) 1 μM Pep1 for 18 h, the roots were stained with 30 μM H_2_DCF-DA solution for 10 min and photographed under fluorescence microscopy (Olympus, BX53). Bars = 100 μm. (**C**) The statistical analysis of ROS production in (**B**). Data are means ± SD from three independent experiments. (*n* = 24). (**D**) Quantitative RT-PCR analysis of mRNA levels of *PROPEP1* in the wild type roots treated with or without (Control) DC3000 solution (initial OD_600_ of 0.02) or 1 μM Pep1 for 4h, respectively. The expression level of control was set as 1.0. Data are means ± SD (*n* = 3). (**E**) H_2_DCF-DA staining of H_2_O_2_ in WT, *pepr1*, *pepr2* and *pepr1 pepr2* roots. Six-day-old seedlings were incubated in half-strength MS liquid medium supplemented with 1 μM Pep1 for 18 h, the roots were stained with 30 μM H_2_DCF-DA solution for 10 min and photographed under fluorescence microscopy (Olympus, BX53). Bars = 100 μm. (**F**) The statistical analysis of ROS production in WT, *pepr1*, *pepr2* and *pepr1 pepr2* roots, Six-day-old seedlings were incubated in half-strength MS liquid medium supplemented with or without 0.1 and 1 μM Pep1 for 18 h, the roots were stained with 30 μM H_2_DCF-DA solution for 10 min and photographed to analyze the ROS production. Data are means ± SD from three independent experiments. (*n* = 25). The relative ROS production of each treatment in (**C**,**F**) was normalized to control of wild type roots (100%). (**G**) Quantitative RT-PCR analysis of mRNA levels of *PEPR1*, *PEPR2* and *PROPEP1* in the wild type roots treated with or without (Control) 10 mM H_2_O_2_ for 4 h. The expression level of control was set as 1.0. Data are means ± SD (*n* = 3). Asterisks in (**C**,**D**,**F**) and (**G**) indicated statistically significant differences compared with the controls in each genotype (Tukey’s test, * *p* < 0.05, ** *p* < 0.01, *** *p* < 0.001).

The DAMPs Pep1 is released from the plant cells and is perceived by its two receptors, PEPR1 and PEPR2 to elicit immune responses during pathogen or wounding induction [11]. Our previous results showed that the Pep1–PEPR signaling pathway functions in plant leaf to defend against pathogen invasion by triggering stomatal closure [12]. In this study, we showed that PEPRs were required for the immunity responses in roots. To investigate whether Pep1–PEPRs system functions in roots to regulate the immunity response, we treated seedlings with Pep1 and detected the ROS levels in the roots. We found that Pep1 treatment effectively enhanced the production of O_2_^−^ and H_2_O_2_ in wild type roots, as shown in Figure 2A–C. As Pep1 is derived from the precursor protein, called PROPEP1, which is transcriptionally induced by pathogens or wounding to amplify the leaf immunity [30,31,32]. We continually investigated the root mRNA level of *PROPEP1* in the wild type roots by stimulation with *Pst* DC3000 suspension. As expected, the expression of *PROPEP1* was induced by *Pst* DC3000 treatments, as shown in Figure 2D. Interestingly, the mRNA level of *PROPEP1* the in roots was also strongly induced by Pep1, as shown in Figure 2D. Taken together, these results suggest that the Pep1–PEPRs system functions in roots to mediate root immunity.

To further dissect the functions of PEPRs in the Pep1 signal of roots, we applied 0.1 and 1 μM Pep1 solution to wild type, *pepr1*, *pepr2,* and *pepr1 pepr2* roots to observe ROS production. No significant difference in ROS accumulation appeared between the wild type and mutant roots grown in normal conditions, as shown in Figure 2F. Under Pep1 treatment, the roots’ ROS production was largely dependent on the presence of *PEPR2*, as the seedlings mutated in PEPR2 (*pepr2, pepr1 pepr2*) were essentially insensitive *t*o Pep1 treatment, as shown in Figure 2E,F. Although the PEPR1 receptor may also contribute to the perception of Pep1 signals, such a contribution only occurred at 1 μM of Pep1 treatment, as the *pepr2* roots were shown to change the Pep1 sensitivity at this concentration, as shown in Figure 2E,F. These results suggest that *PEPR2* plays a dominant role in the roots to respond the Pep1-induced immune signaling, while *PEPR1* emerges as a major player in Pep1 perception in leaf immunity [12].

In leaves, the positive feedback regulation of immunity signaling from downstream ethylene or jasmonic acid signaling to Pep1–PEPR1 contributes to the efficient protection mechanism to enhance defense ability [33]. To investigate whether ROS signaling plays a positive role in the regulation of Pep1-PEPR signaling in roots, we treated the wild type roots with H_2_O_2_ solution and analyzed the mRNA level of the *PEPRs* and *PROPEP*1. We found that *PEPR1*, *PEPR2,* and *PROPEP1* were all transcriptional induced by H_2_O_2_, as shown in Figure 2G. Taken together, these results suggest that ROS signaling up-regulates the expression of both the Peps and their receptors, forming a positive feedback loop, which is critical to the overall robustness of root immunity.

### 2.3. RBOHD and ROBHF Are Required for Pep1-Induced ROS Production

**Figure 3:**
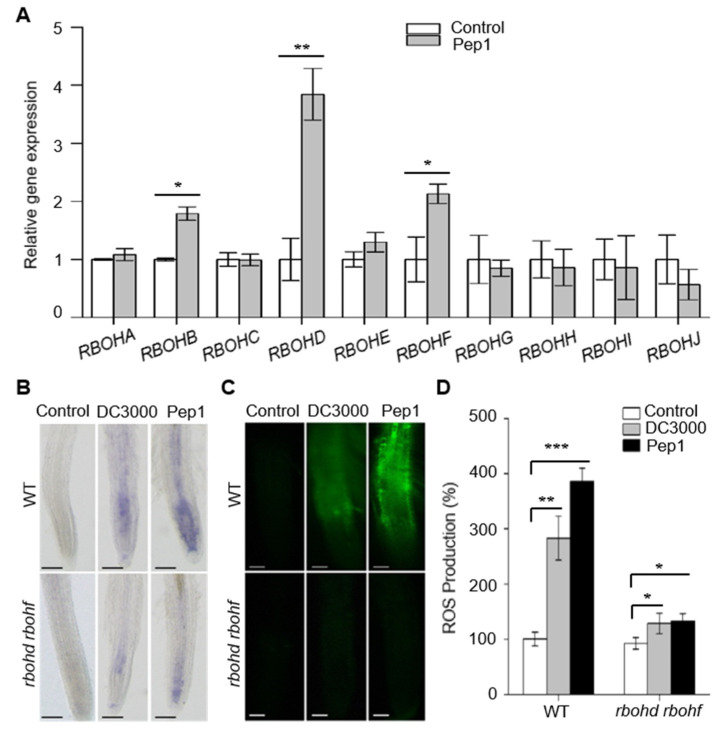
RBOHD and RBOHF are required in pathogen and Pep1-induced ROS production. (**A**) The transcriptional level of RBOHs in wild type (WT) roots upon Pep1 treatment. Two-week-old WT seedlings were incubated in half-strength MS liquid medium with or without (control) 1 μM Pep1 for 4 h. The expression level of control (0 h) was set as 1.0 and Pep1 treatment levels were normalized to the control level. Data are means ± SD (*n* = 3). (**B**) NBT staining of superoxide (O_2_^−^) anion in WT and *rbohd rbohf* roots. Bars = 100 μm. (**C**) H_2_DCF-DA staining of H_2_O_2_ in WT and *rbohd rbohf* roots. Bars = 100 μm. In (**B**,**C**), the six-day-old seedlings were incubated in half-strength MS liquid medium (control) or medium with DC3000 (initial OD_600_ of 0.02) or Pep1 (1 μM) added for 18 h. (**D**) The statistical analysis of the ROS production in WT and *rbohd rbohf* roots as shown in (**C**). Data are means ± SD from three independent experiments. (*n* = 30). The relative ROS production of each treatment was normalized to the control of WT roots (100%). Asterisks in (**A**,**D**) indicate statistically significant differences compared with the controls (Tukey’s test, * *p* < 0.05, ** *p* < 0.01, *** *p* < 0.001).

In *Arabidopsis*, the NADPH oxidases, also known as RBOHs, are major ROS-generating enzymes [25]. To test the function of RBOHs in Pep1 signaling, we first analyzed the transcriptional levels of *RBOHs* family upon Pep1 treatment. Among the ten members of *RBOH* family, the expression of *RBOHD* and *RBOHF* were significantly induced, while expression of *RBOHB* was slightly enhanced by Pep1 in root, as shown in Figure 3A, suggesting that RBOHD and RBOHF function vitally in the root to respond the Pep1 signaling. As RBOHD and RBOHF are the two major enzymes involved in H_2_O_2_ synthesis during plant defense against biotic stress, we challenged the *rbohd rbohf* double mutant seedlings with *Pst* DC3000 and Pep1 treatment and found that the ROS production was increased compared to the mock control, but the level was much lower than wild type as shown in Figure 3B-D. Taken together, these results suggest that RBOHD and RBOHF are required to produce ROS and regulate the root immune response, in agreement with previous results that the two genes played key roles in PAMPs- and DAMPs-induced immune responses in leaf tissues [21,24,34].

### 2.4. Pep1 Signaling Intersects with ROS Signaling Pathway to Inhibit the Root Growth

**Figure 4:**
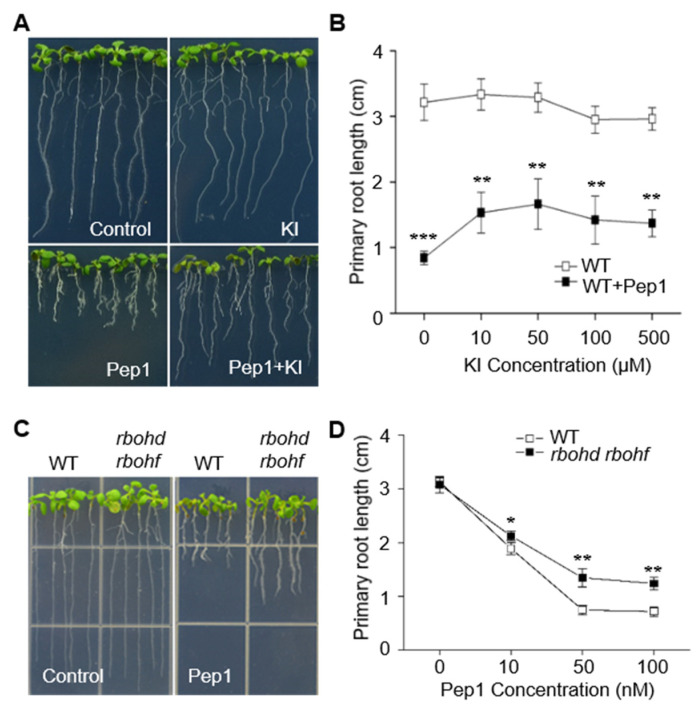
Pep1 intersects with ROS signaling to regulate root growth. (**A**) The growth phenotype of the wild type (WT) roots. The three-day-old seedlings were transplanted on half-strength MS agar medium supplemented with or without (control) 50 μM KI, 100 nM Pep1, or 100 nM Pep1 +50 μM KI for six days. (**B**) The statistical analysis of the primary root length in WT plants. The three-day-old seedlings were transplanted on half-strength MS agar medium, supplemented with or without 100 nM Pep1 in the presence of various concentrations of KI (0–500 μM) for six days. Data are means ± SD from three independent experiments (*n* = 15). (**C**) The growth phenotype of WT and *rbohd rbohf* root. The three-day-old seedlings were transplanted on half-strength MS agar medium supplemented with or without (control) 100 nM Pep1 for six days. (**D**) The statistical analysis of the ROS production in WT and *rbohd rbohf* roots. Six-day-old seedlings were incubated in half-strength MS liquid medium with a various concentration of Pep1 (0-100 nM) for six days. Data are means ± SD from three independent experiments (*n* = 15). Asterisks in (**B**,**D**) indicate statistically significant differences compared with the WT in each treatment (Tukey’s test, * *p* < 0.05, ** *p* < 0.01, *** *p* < 0.001).

The application of elicitor peptides to plants not only enhances plant immunity, but also impairs plant development. Our previous results indicated that Pep1 induced a strong inhibition in root growth [13]. The strong induction of ROS by the Pep1–PEPRs system in roots raises a question of whether this event causes the root growth inhibition, as the application of exogenous H_2_O_2_ was also shown to inhibit root growth, as shown in Figure S3, in agreement with the previous study [29]. To figure this out, we treated the seedling roots with Pep1 in the presence of various concentrations of KI. As shown in Figure 4A–B, the addition of KI significantly undermined the Pep1 effect on root growth inhibition, with 50 μM KI achieving the maximum mitigation, suggesting that Pep1 intersects with ROS signaling to regulate root growth. We further detected the root growth in *rbohd rbohf* double mutants upon Pep1 treatment and found that the double mutant decreased the Pep1 sensitivity, as shown in Figure 4C–D, suggesting that RBOHD and RBOHF are required for Pep1-regulated root growth. Taken together, these data suggest that Pep1, in turn, activates the ROS signaling to inhibit root growth.

### 2.5. BIK1 Is Required to Regulate Pep1 Signaling in Roots

**Figure 5:**
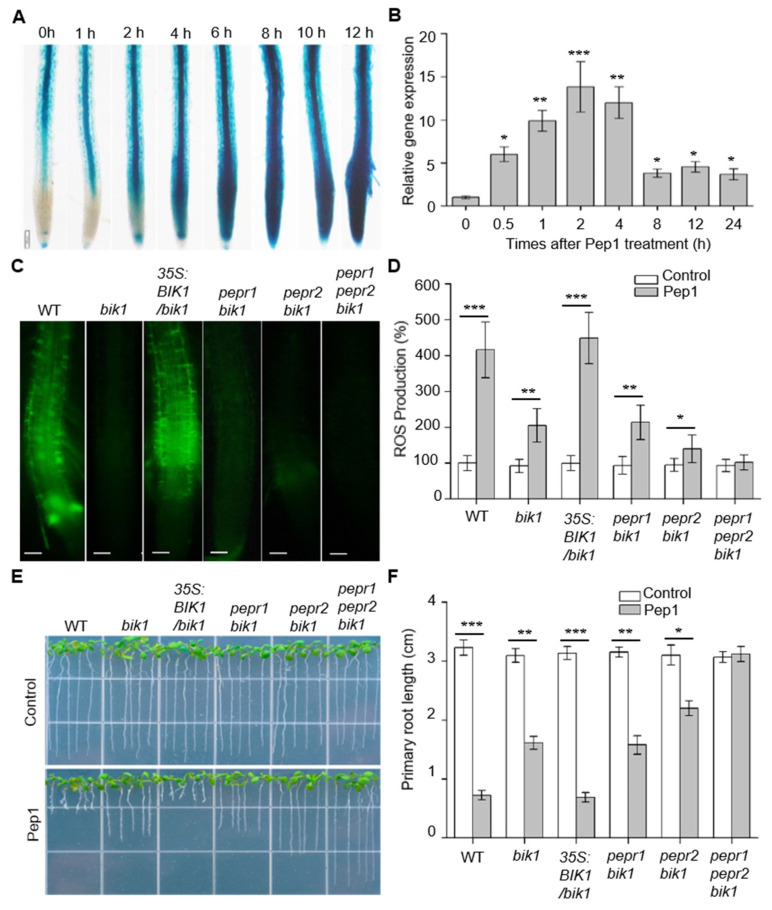
BIK1 is required to regulate Pep1-induced ROS production and root growth inhibition. (**A**) Histochemical staining of GUS activity in the roots of transgenic plants harboring *proBIK1:GUS* upon exposure to Pep1. The six-day-old plants were transferred on half-strength MS agar medium, supplemented with 1 μM Pep1 for 1, 2, 4, 6, 8, 10, and 12 h, respectively. The experiments were repeated three times with similar observations. Bars = 200 μm. (**B**) qRT-PCR analysis of mRNA levels of *BIK1* in the wild type (WT) roots. The two-week-old plants were incubated in half-strength MS liquid medium, supplemented with 1 μM Pep1 for 0.5, 1, 2, 4, 8, 12, and 24 h, respectively. The expression level of control (0 h) was set as 1.0 and the Pep1 treatment levels were normalized to the control level. Data are means ± SD (*n* = 3). (**C**) H_2_DCF-DA staining of H_2_O_2_ in WT, *bik1*, *35S:BIK1*/*bik1, pepr1 bik1*, *pepr2 bik1,* and *pepr1 pepr2 bik1* roots. Six-day-old seedlings were incubated in half-strength MS liquid medium, supplemented with 1 μM Pep1 for 18 h. The roots were stained with 30 μM H_2_DCF-DA solution for 10 min and photographed under fluorescence microscopy (Olympus, BX53). Bars = 100 μm. (**D**) The statistical analysis of ROS production, as indicated in (**C**). Data are means ± SD from three independent experiments. (*n* = 30). The relative ROS production of each treatment was normalized to control of wild type roots (100%). (**E**) The growth phenotype of WT, *bik1*, *35S:BIK1*/*bik1, pepr1 bik1*, *pepr2 bik1,* and *pepr1 pepr2 bik1* roots. The three-day-old seedlings were transplanted on half-strength MS agar medium supplemented with or without (control) 100 nM Pep1 for six days. (**F**) The statistical analysis of the primary root length, as indicated in (**E**). Data are means ± SD from three independent experiments (*n* = 15). Asterisks in (**B**,**D**,**F**) indicate statistically significant differences compared with the untreated controls in each genotype (Tukey’s test, * *p* < 0.05, ** *p* < 0.01; *** *p* < 0.001).

The botrytis-induced kinase 1 (BIK1) functions downstream of the Pep1–PEPR1 system to mediate ROS production and leaf immunity [21,24,28]. BIK1 interacts with and phosphorylates the RBOHD to initiate ROS production [21]. To investigate whether BIK1 is required to regulate Pep1–PEPRs signaling in root tissues, firstly, we constructed the *proBIK1:GUS* transgenic plants to analyze the expression pattern of *BIK1* under Pep1 treatment. The GUS staining analysis showed that the *BIK1* promoter conferred relatively high signals in the vascular tissues of the root elongation and maturation zone, but not in the meristematic zone, as shown in Figure 5A, in agreement with the previous results [35]. Under the Pep1 treatments, the GUS staining signals appeared at the meristematic zone within 2 h, and the staining intensities were gradually enhanced along the Pep1 treatment, as shown in Figure 5A. Because GUS activity is stable over a long period of time, it is not suitable for measuring the kinetics of gene expression over a time course. We, therefore, performed qRT-PCR to measure the native mRNA levels of the *BIK1* over a time course upon Pep1 treatment. As shown in Figure 5B, the mRNA levels of *BIK1* in roots were strongly increased after exposure to 100 nM Pep1, consistent with the GUS assay results. These data indicate that the expression of *BIK1* is induced by Pep1 signaling in plant root.

In order to get more information on BIK1 function in Pep1-induced ROS production and root growth inhibition, we performed Pep1-induced ROS production and root growth assay on *bik1* mutants. The function loss of BIK1 decreased the Pep1 sensitivity both in root ROS accumulation and growth inhibition, whereas *bik1* mutant expressing *BIK1,* driven by *CaMV 35S* promoter (*35S:BIK1*/*bik1*), became sensitive to Pep1 treatment, as shown in Figure 5C–F. Furthermore, we crossed *bik1* with *pepr1*, *pepr2,* or *pepr1 pepr2* double mutant to obtain *pepr1 bik1, pepr2 bik1* double, and *pepr1 pepr2 bik1* triple mutants, respectively, as shown in Figure S4. Under half-strength MS agar medium growth conditions, no significant difference was observed among these multiple mutants and the *bik1* mutant, as shown in Figure 5E–F. However, when Pep1 was supplemented in the medium, the *pepr1 bik1* double mutant showed similar sensitivity to the *bik1* mutant, both in ROS production and root growth, whereas those Pep1-induced responses in the *pepr2 bik1* and *pepr1 pepr2 bik1* mutants were further decreased or even disappeared, respectively, as shown in Figure 5C–F. These results further prove that PEPR2 plays a predominant role in root Pep1 perception. BIK1 functions downstream of PEPR2 to regulate Pep1 signaling in the roots. Moreover, we also found that the disruption of the PBL1, a homologous protein of BIK1, was shown to decrease the Pep1 sensitivity in root ROS accumulation and growth inhibition. The *bik1 pbl1* double mutant further reduced the Pep1 sensitivity, as shown in Figure S5. These results consistently show that multiple BIK1 family members are involved in Pep1-induced defenses [28].

**Figure 6:**
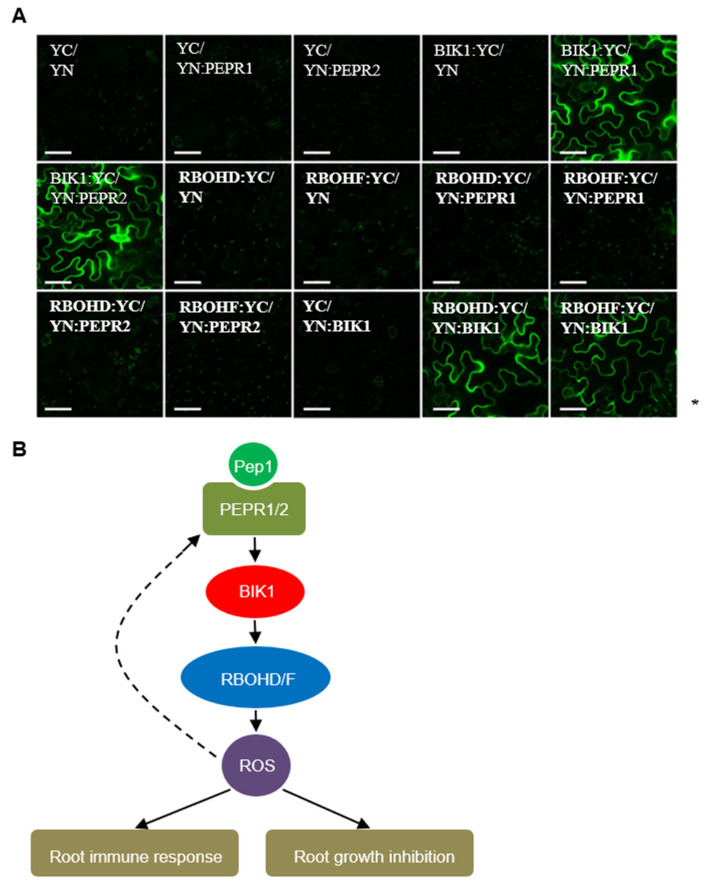
BIK1 interacts with PEPR1/2 and RBOHD/F. (**A**) Bimolecular fluorescence complementation of YC/YN, YC/YN:PEPR1, YC/YN:PEPR2, BIK1:YC/YN, BIK1:YC/YN:PEPR1, BIK1:YC/YN:PEPR2, RBOHD:YC/YN, RBOHF:YC/YN, RBOHD:YC/YN:PEPR1, RBOHF:YC/YN:PEPR1, RBOHD:YC/YN:PEPR2, RBOHF:YC/YN:PEPR2, YC/YN:BIK1, RBOHD:YC/YN:BIK1 and RBOHF:YC/YN:BIK1. Images were taken using confocal laser scanning microscopy. Bars = 50 μm. (**B**) A proposed model of the Pep1–PEPRs signaling pathway activates the ROS signaling in roots.

To confirm the connection between BIK1 and PEPRs, we performed bimolecular fluorescence complementation (BiFC) experiments by transforming the YN:PEPR1 or YN:PEPR2 plasmid with the BIK1:YC plasmid into tobacco leaves. In addition to PEPR1, we found that PEPR2 could also directly interact with BIK1, as shown in Figure 6A. These results are consistent with the notion that both PEPR1 and PEPR2 interact with BIK1 to regulate plant immunity. The PEPR1-BIK1 component is the main focus on leaves, whereas the PEPR2-BIK1 component functions in roots to regulate root immunity and development. Furthermore, we continually analyzed the relationship between PEPRs or BIK1 with RBOHD/F. We transformed the YN:PEPRs or YN:BIK1 plasmid with the RBOHD:YC or RBOHF:YC plasmid into tobacco leaves, and found that BIK1, but not PEPRs, were shown to interact with RBOHD and RBOHF, suggesting that BIK1 should be a central hub to connect the PEPRs and RBOHD/F, as shown in Figure 6A.

## 3. Discussion

The plant innate immune system is initiated by the perception of PAMPs or DAMPs via hosts’ PRR and has been widely studied in plant leaves. In contrast to leaves, the root system faces more serious threats from the rhizosphere pathogenic microorganism. It is possible that roots may develop complex mechanisms to detect these pathogens carrying PAMPs or stimulate the immune response through its own DAMPs to defend against pathogens. However, relatively little is known about the immunity responses in roots. In this study, we show that *Arabidopsis* Pep1 and its receptors, PEPRs, play a vital role in regulating the root immune responses under pathogen threats. The perception of Pep1 by PEPRs triggers strong ROS production by activating the BIK1-RBOHD/F component in the roots. These findings reveal a previously unrecognized signaling pathway by which danger peptides regulate the roots’ immune responses.

The results in this work indicate that PEPRs are required in root defense systems. The loss-of-function of *PEPRs* impaired root ROS production upon *Pst* DC3000 infection, as shown in Figure 1. These results are consistent with the responses in leaf tissue, suggesting that the PEPRs-dependent activation of immune responses are commonly shared between both root and leaf tissues [30,34,36]. Among the two receptors for Pep1, PEPR1 and PEPR2 appear to function divergently in different tissues. PEPR1 plays a more important role than PEPR2 in mediating the Pep1-induced defense responses in leaves [12]. In our study, we proposed that PEPR2 functioned more significant than PEPR1 to perceive the Pep1 in the roots, as shown in Figure 2. The diverging function of these two genes could not be concluded by organizational expression differences, as both the PEPR1 and PEPR2 are expressed similarly in roots and leaves [10,12,13]. The different downstream components of the two receptors may contribute to the function difference; however, the molecular basis for this is not delineated and demands a more in-depth examination.

The application of danger peptides not only activates the roots’ immune responses, but also inhibits root growth. In the previous study, we proved that the Pep1–PEPRs system intersected with auxin signaling to regulate root growth [13]. In this study, we found that Pep1 also intersected with ROS signaling to induce root growth inhibition, as the blocking of the ROS signaling pathway mimicked the Pep1 effect on root growth, as shown in Figure 4. However, interfering with auxin or ROS signaling separately could not completely mimic the Pep1 effect on root growth, suggesting that multiple signaling pathways may be activated by Pep1 signaling. Whether those multiple signals function relative to one another or separately demands more in-depth examination.

The NADPH oxidases, also known as RBOH proteins, provide localized ROS bursts to regulate growth, developmental processes, and stress responses in plant cells [22,23]. The RBOH members function differently in response to environmental stimuli or cell growth signals. RBOHD and RBOHF have been reported to function vitally in leaves to mediate ROS production under the induction of pathogens, PAMPs, or DAMPs [21,24,37]. In the current study, we found that the activation of Pep1–PEPR triggered a strong ROS accumulation in the roots. RBOHD and RBOHF attributed to root ROS production upon Pep1 induction, suggesting that the two synthetases function vitally both in leaves and roots to mediate immunity responses. In addition to RBOHD and RBOHF, we found that the mRNA level of RBOHB was also induced by Pep1 treatment, as shown in Figure 3. RBOHB expresses in the whole tissues of plant and regulates seed development [38,39]. Our finding that RBOHB is induced by Pep1 signaling helps to provide a new insight that RBOHB may also function in roots to regulate the immune responses, but further examination needs to be done in future research.

PRRs interact with other components to activate immune responses. The plasma–membrane-associated kinase BIK1, interacts directly with PRRs, such as FLS2 and EFR, in plant cells [40,41]. BIK1 also acts as a direct substrate of these PRRs. The perception of PAMPs to PRRs results in the rapid phosphorylation of BIK1, which is then released from the PRRs to activate downstream signaling [40,41]. PEPR1 has been reported to directly interact with and phosphorylate BIK1 to regulate leaf immunity [28]. In the current study, we found that the loss-of-function mutant of *bik1* was shown to mimic the Pep1 effect on root ROS accumulation and root growth inhibition, as shown in Figure 5. BIK1 also interacted with PEPR2 in vivo, as shown in Figure 6. These results provide an evidence that BIK1 may acts as a direct substrate of PEPR2 to regulate Pep1 signaling in the roots. The activated BIK1 further interacts with and phosphorylates the RBOHD at the S39, S339, and S343 sites of the N terminus, which plays a critical role in the PAMP- and DAMP-induced ROS burst [21]. In addition to RBOHD, the RBOHF also contributes to ROS production to regulate plant immunity [34,39]. We found that BIK1 also directly interacted with RBOHF in vivo, as shown in Figure 6. Through protein interaction analysis, we provide evidence that BIK1 may interact with and phosphorylate the RBOHF in a similar way to RBOHD to trigger ROS production, but the potential phosphorylation sites need to be further explored. According to previous research and our results, we propose a model of the Pep1–PEPRs signaling pathway in activating the ROS signaling in roots—the binding of Pep1 to PEPRs, leading to the phosphorylation and activation of BIK1 and the activated BIK1 continually interacts with and phosphorylates the RBOHD/F to activate ROS production, which further inhibits root growth and regulates root immunity, as shown in Figure 6B. Besides being used as a weapon against pathogens, the ROS somehow serve as molecular signals to positively regulate the Pep–PEPR pathway.

In conclusion, the results of this study have uncovered a significant role of Pep1-PEPRs in root immune regulation, Meanwhile, we also revealed the genetic mechanism by which the Pep1–PEPR2 system regulates root immunity and root growth. It will be interesting to further investigate the difference between PEPR1 and PEPR2 function in roots and leaves through detailed analysis of the signaling components that connect with the Pep1–PEPRs pathway.

## 4. Materials and Methods

### 4.1. Plant Materials and Growth Conditions

*Arabidopsis thaliana* mutant lines *pepr1-2*, *pepr2-2*, and *pepr1-2 pepr2-2* double mutant [9,42], *bik1, 35S:BIK1/bik1*, *pbl1*, *bik1 pbl1*, and *rbohd rbohf* [24,28], were described in previous studies. *pepr1 bik1*, *pepr2 bik1*, and *pepr1 pepr2 bik1* mutants were obtained by genetically crossing *pepr1*, *pepr2*, or *pepr1 pepr2* with *bik1*, respectively. The double and triple mutants were identified by PCR and RT-PCR using primers described in Table S1. All *Arabidopsis* lines in this study were Columbia (Col-0) ecotype background. The seedlings were grown on half-strength Murashige and Skoog (MS) medium, containing 1% sucrose and 0.8% phytogel (Sigma-Aldrich, St. Louis, MO, USA) in the growth chamber. The growth conditions had 90 μmol/m^2^/s light intensity with a 16 h light/8 h dark photoperiod at 22 °C.

### 4.2. Plasmid Constructions

For transgenic *proBIK1:GUS* lines, the promoter region, upstream of the starting codon of *BIK1,* was amplified and cloned into the pCAMBIA1300 fused GUS binary vector. The constructs were transformed into *Agrobacterum tumefaciens* strain GV3101 and further transformed into the wild type *Arabidopsis*. For bimolecular fluorescence complementation (BiFC) constructs, the two BiFC vectors pSPYCE(M) and pSPYNE(R)173 were cut with Hind III and EcoR I restriction enzymes to generate the 35S-MCS-eYFP_C155_-NosT and 35S-eYFP_N173_-MCS_2_-NosT fragments. The *PEPR1*, *PEPR2*, *BIK1, RBOHD,* and *RBOHF* coding sequences without stop codons were fused to the above two fragments and cloned into the pCAMBIA-1300 binary vector. The constructs were transformed into *Agrobacterum tumefaciens* strain GV3101 and further transformed the corresponding *Arabidopsis* lines by floral-dip method [43]. The primers used for constructions are listed in Table S1.

### 4.3. Bacterial Strains and Infections

For the seedlings infected with *Pseudomonas syringae* p.v. tomato DC3000 (*Pst* DC3000), the bacterial strain was cultured overnight at 28 °C in Luria–Bertani (LB) liquid medium supplemented with 50 μg/mL rifampicin. The bacterial strains were centrifuged, washed three times with water, and then resuspended with half-strength MS liquid medium to an initial OD_600_ of 0.02 to infect the seedlings.

### 4.4. ROS Detection

The seedlings were incubated in 12-well cell culture plates with bacterial suspensions (initial OD_600_ of 0.02), 1 μM Pep1 added with or without 100 μM KI for 18 h to detect the root ROS production. For ROS detected with NBT (nitroblue tetrazolium) staining, the seedlings were incubated in 1 mM NBT solution (20 mM Potassium phosphate buffer, pH 6.2; 0.1 M NaCl) at room temperature in the dark for 1 h. The roots were washed three times with water and photographed under the fluorescence microscopy (BX53, Olympus, Tokyo, Japan). For ROS detected with 2′,7′-dichlorofluorescein diacetate (H_2_DCF-DA) (Sigma-Aldrich, St. Louis, MO, USA), the previously described process was performed [44] with a slight modification. In brief, the seedlings were incubated in 30 μM H_2_DCF-DA solution for 10 min in darkness. The roots were then washed three times with water and photographed under fluorescence microscopy (BX53, Olympus, Tokyo, Japan) equipped with a camera (DP72, Olympus, Tokyo, Japan). The objective lens operated at 20X, the wavelength range of the light irradiation was 460–490 nm and the power density was about 100 mW/cm^2^. The fluorescence intensities in the roots were quantified as average pixel intensities by using Image J 1.51K Software (National Institutes of Health, Bethesda, MD, USA).

### 4.5. Histochemical GUS Analysis

GUS activity was analyzed by histochemical staining, as previously described [45] with slight modifications. Briefly, the T_2_ transgenic seedlings were incubated in GUS staining solution (2 mM 5-bromo-4-chloro-3-indolyl-*β*-d-glucuronide, 1 mM K_3_(Fe(CN)_6_), 1 mM K_4_Fe(CN)_6_·3H_2_O, 10 mM Na_2_EDTA, 0.1% Triton X-100, and 50 mM Na_3_PO_4_, pH 7.0) at 37 °C for 6 h. The seedlings were decolorized with 75% (*vol/vol*) ethanol for 24h and then photographed under the light microscope (SZX16, Olympus, Tokyo, Japan) and equipped with a camera (DP72, Olympus, Tokyo, Japan).

### 4.6. Quantitative RT-PCR Analysis

Total RNA in leaves and roots were extracted using the TRIzol reagent (Invitrogen, Carlsbad, CA, USA), according to the manufacturer’s protocol. The 2 μg RNA was used to synthesis the cDNA by using M-MLV Reverse Transcriptase (Promega, Madison, WI, USA). qRT-PCR analysis was performed using the FastStart Universal SYBR Green mastermix (Roche Diagnostics, Hong Kong) on a CFX Connect Real Time System (Bio-Rad, Berkeley, CA, USA) using *Actin2* as internal standards. The primers of the target gene used are listed in Supplemental Table S1.

### 4.7. BiFC Analysis

The BiFC constructs were analyzed after the infiltration of the *N. benthamiana* leaves, as described earlier [46]. Briefly, the Agrobacterium strains contained the BiFC constructs and p19 silencing plasmids were cultured until the OD_600_ arrived. Then, 1.0 Agrobacterium strains were centrifuged and resuspended with 10 mM MgCl_2_ to a final OD_600_ of 0.5. We then mixed the two analyzed BiFC Agrobacterium strains with p19 Agrobacterium strain in a ratio of 1:1:1. Then, the three strains in the *N. benthamiana* leaves were co-infected and cultured for 2 days. The tobacco leaves were then photographed under the Leica TCS SP8 confocal microscope (Leica, Weztlar, Germany) with the excitation wave length of 488 nm.

### 4.8. Root Growth Analysis

To analyze the root growth, the three-day-old seedlings grown on the normal 1/2 MS medium were transferred to the half-strength MS medium, supplemented with or without 100 nM Pep1 and the various concentrations of KI or H_2_O_2_ and were treated for six days. The plants were then collected for photographing and measuring.

### 4.9. Statistical Analysis

For all the experiments, three independent repetitions were performed. One-way ANOVA Tukey’s test was used for statistical analysis. Asterisks in the figures denote significant differences, as follows: * *p* < 0.05, ** *p* < 0.01, and *** *p* < 0.001.

## Supplementary Materials

Supplementary materials can be found at https://www.mdpi.com/1422-0067/21/13/4590/s1.
