# Supplementary material for: Danger-Associated Peptide Regulates Root Immune Responses and Root Growth by Affecting ROS Formation in Arabidopsis
Source: Int J Mol Sci. 2020 Jun 28;21(13):4590. doi: 10.3390/ijms21134590 (PMC7369728; doi:10.3390/ijms21134590)
Supplement: Supplementary file 1 [file ijms-21-04590-s001.pdf]

Supplementary data

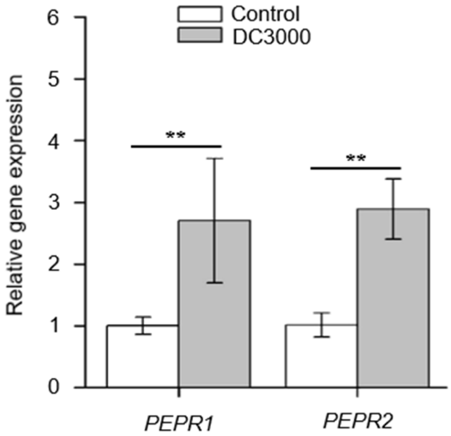

Supplementary figure 1. PEPR1 and PEPR2 are transcriptional induced by *Pst.*DC3000 in leaves.

Two-weeks-old wild type seedlings were incubated in *Pseudomonas syringae* p.v. tomato DC3000 solution (initial OD_{600} of 0.02) for 4h. The expression level of control (0 h) was set as 1.0 and DC3000 treatment levels were normalized to the control level in each ecotype. Data are means ± SD (n = 3). Asterisks indicated statistically significant differences compared with the controls in each gene (Tukey's test, **P <0.01).

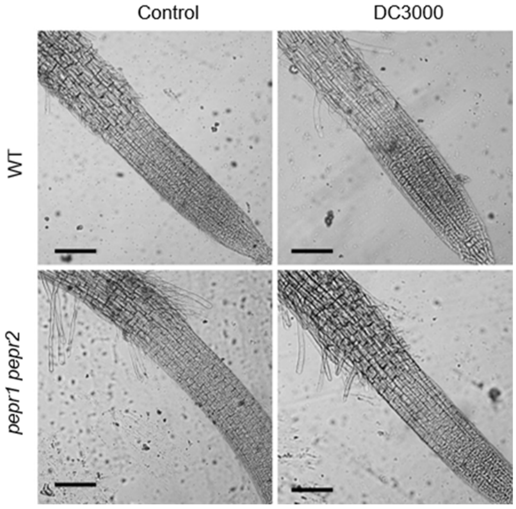

Supplementary figure 2. The root structure in wild type and *pepr1 pepr2* seedlings.

Six-day-old wild type (WT) and *pepr1 pepr2* seedlings were incubated in half-strength MS liquid medium (Control) or DC3000 solution (initial OD600 of 0.02)

for 18 h, the roots were photographed under microscopy (Olympus, BX53). Bars = 100 μm.

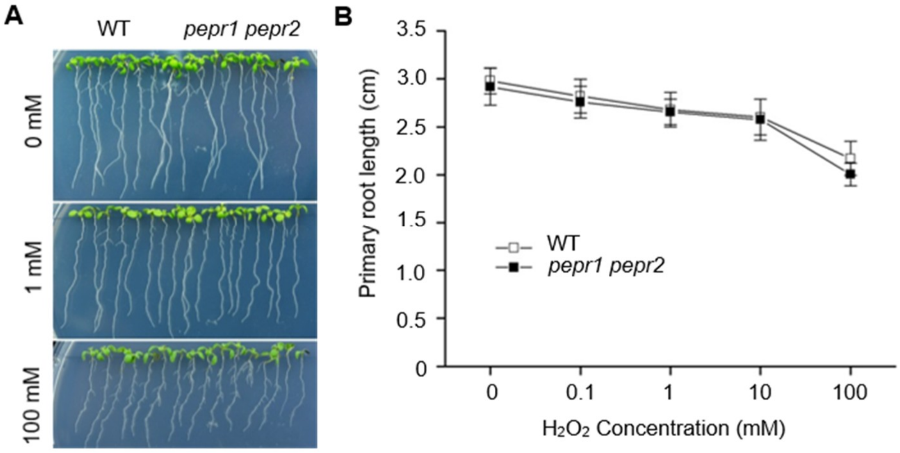

Supplementary figure 3. H_2O_2 inhibits the roots growth.

(A) The growth phenotype of wild-type (WT) and *pepr1 pepr2* mutants. The 3-day-old seedlings were transplanted on half-strength MS agar medium supplemented with 0, 1, and 100 mM H_2O_2 for 6 days. (B) The statistical analysis of the primary root length of WT and *pepr1 pepr2* mutants. The 3-day-old seedlings were transplanted on half-strength MS agar medium supplemented with various concentrations of H_2O_2 (ranged from 0-100 mM) for 6 days. Data are means ± SD from 3 independent experiments (n = 16).

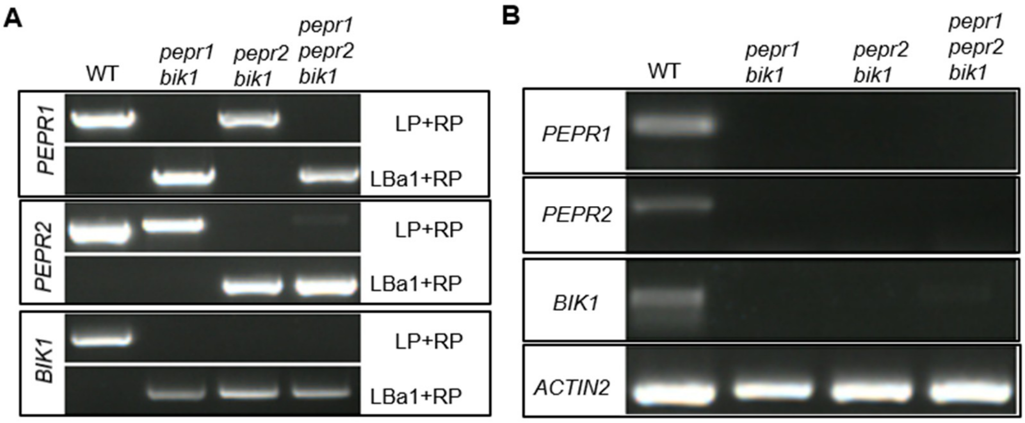

Supplementary figure 4. The identification of *pepr1 bik1, pepr2 bik1 and pepr1 pepr2 bik1* mutants.

(**A**) Confirmation of the T-DNA insertion in the *pepr1 bik1, pepr2 bik1 and pepr1 pepr2 bik1* mutants by PCR. (B) RT-PCR detection of the transcriptional level of *PEPR1, PEPR2* and *BIK1* expression in wild type, *pepr1 bik1, pepr2 bik1 and pepr1 pepr2 bik1* plants.

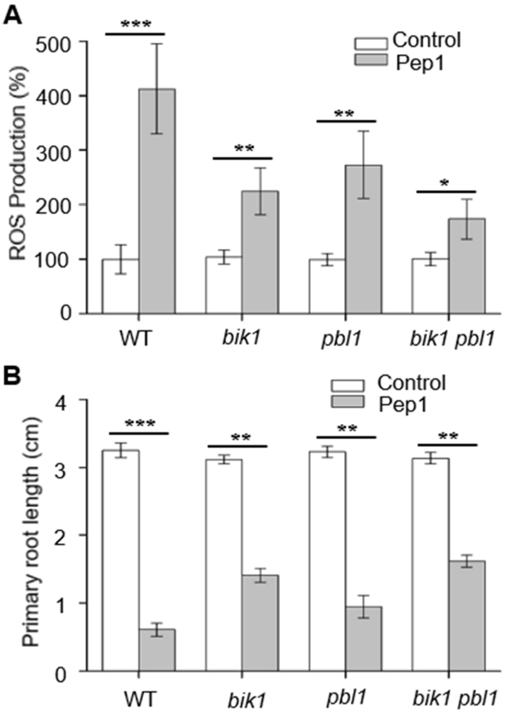

Supplementary figure 5. BIK1 with its homolog PBL1 are required in Pep1 signaling pathway

(A) The statistical analysis of ROS production in WT, *bik1*, *pbl1* and *bik1 pbl1* roots. Six-day-old seedlings were incubated in half-strength MS liquid medium with or without (Control) 1 µM Pep1 for 18 h, the roots were stained with 30 µM H_2DCF-DA solution for 10 min and analyze the ROS production. Data are means ± SD from three independent experiments. (n = 25). The relative ROS production of each treatment was normalized to control of wild type roots (100%). (B) The statistical analysis of the primary root length of WT, *bik1*, *pbl1* and *bik1 pbl1* mutants. The 3-day-old seedlings were transplanted on half-strength MS agar medium supplemented with or without (Control) 100 nM Pep1 for 6 days. Data are means ± SD from 3 independent experiments (n = 10). Asterisks in (A) and (B) indicate statistically significant differences compared with the controls in each genotype (Tukey's test, *P <0.05; **P <0.01, ***P <0.001).

Supplementary Table 1 Primers used in this study

Primer name	Primer sequences (5'-3')
(1) qRT-PCR analyze	
Actin2-F	CTGTTCTCTCCTTGTACGCCAGT
Actin2-R	CGGGTAATTCATAGTTCTTCTCGAT
BIK1-F	CACTCTCACTCCGACTAAAC
BIK1-R	ACGGTGTTCATCTTCTAAGC
PEPR1-F	ACCTGGCAAATCGTGCTAAT
PEPR1-R	TTCCTCCTGAGTGAAGACATAA
PEPR2-F	GCGTCTATTCGTCAGTGTAA
PEPR2-R	AGCTATTGCTTCCGAGGTT
PROPEP1-F	CGAAACAGCCGAAGGAGGAA
PROPEP1-R	GGACGGCCTGAGCTAACTTT
RBOHA-F	CGTGTCATGTCCCATTTCGC
RBOHA-R	TTCACTAACCCAGCTGCTCC
RBOHB-F	CCGGGGATGATTACCTCAGC
RBOHB-R	AGGGAAGTTGACAAACCTTGGA
RBOHC-F	CGGCAGGAGTTAGTGGTCTG
RBOHC-R	TTGGTGTGGCTCCAATCCC
RBOHD-F	ACTCTCCGCTGATTCCAACG
RBOHD-R	ATCGCCGGAGACGTTATTCC
RBOHE-F	AAGACCTCGTCATGTGGTTCA
RBOHE-R	AGAACCCAGCTTCTTTGCCA
RBOHF-F	CTTGGCATTGGTGCAACTCC
RBOHF-R	TCTTTCGTCTTGGCGTGTCA
RBOHG-F	TCATGCATCCAAAACAAGGCA
RBOHG-R	TTCCGCTTGCAGAAGCAATTT
RBOHH-F	GTGCCGATGTTGTTCTACGC
RBOHH-R	TGCAAGAACGTTCCCCGAAT
RBOHI-F	ATGCCCCTGAAGCTGGAAAA
RBOHI-R	CTTCCAATGGTCTTGCGCTG
RBOHJ-F	GCCGACTCACATAAGGTCGT
RBOHJ-R	AGCTCTGCGGTCACTACAAC
**(2) BiFC analysis**	
BIK1-BiFC-F	CGGGATCCATGGGTTCTTGCTTCAGTTCTCG
BIK1-BiFC-R	GGGGTACCCACAAGGTGCCTGCCAAAAG
PEPR1-BiFC-F	CGGGATCCATGAAGAATCTTGGGGGGTTGT
PEPR1-BiFC-R	GGGGTACCCCGAACTGAATCAGAGGAGCA
PEPR2-BiFC-F	GACTAGTATGAGGAATC TTGGGTTACT CGA
PEPR2-BiFC-R	CCCCCGGGTGAACTGAA CCCGAAGTGC
RBOHD-BiFC-F	CGGGATCCATGAAAATGAGACGAGGCAATTC
RBOHD-BiFC-R	GGGGTACCGAAGTTCTCTTTGTGGAAGTC
RBOHF-BiFC-F	GGGGTACCATGAAACCGTTCTCAAAGAACG
RBOHF-BiFC-R	TCCCCCGGGGTGAAATTCAAACTTGGTTGAACC

**(3) Identification of *fls2 pepr1 pepr2* triple mutant**	
LBa1	TGGTTCACGTAGTGGGCCATCG
BIK1-LP	TACTTGGGGCAACTGAGTCAC
BIK1-RP	TTTGTGTAAACCGTTTTTCGG
PEPR1-LP	ACATCAGACGGACGTAAAACG
PEPR1-RP	TGCAATTAGGTGATCCGAAAC
PEPR2-LP	TCCAATGTGAGGCTCCATATC
PEPR2-RP	TTCTCAAAACAAACTCACGGG
**(4) Tissue-specific expression analysis**	
BIK1-GUS-F	ACGCGTCGACTTCCATAAATTGTGGAAAAGTTATC
BIK1-GUS-R	TCCCCCGGGCAAAGCTAAGAACAGATTCGTTTTC
